# Vitality and the Vital Forces

**Published:** 1870-12

**Authors:** H. R. Noel

**Affiliations:** Physiological Department, Baltimore College of Dental Surgery.


AMERICAN JOURNAL

OF

DENTAL SCIENCE.

Vol. IV. THIRD SERIES--DECEMBER, 1870. No. 8.

### ARTICLE I.

### *Vitality and the Vital Forces.*

Lecture by Prof. H. R. NOEL.

Physiological Department, Baltimore College of Dental Surgery.

*Gentlemen* :—In the preceding lecture we have discussed the "Correlation of the forces" in the inorganic world, and have endeavored to demonstrate their mutual convertibility and to show that though we can change the *form* of force, we cannot destroy it; that as man cannot create one atom of matter, so he cannot create one iota of force; and as he cannot destroy matter, neither can he destroy force, for it is "continuous and indestructible"(Grove.)

We wish in the following lecture to show you, that these expressions of force in the inorganic world, and which we have been calling; *motion*, *light*, *heat*, *electricity*, *magnetism* &c., have a most direct and important bearing upon the first principles of a scientific physiology.

We defined physiology to be "the study of the body as a complex machine in full motion" and said that properly it should embrace *first*, *its construction ; second*, *its maintainence ; third*, *the functions of systems and tissues; fourth*, *the laws and conditions of their action.* In construction, or the process called Histogenesis, by which the tissues, organs and systems are evolved, we shall find that these physical forces enter, as an essential element into the initial stage and remain essential until death stops the machinery. We are accustomed to use the terms Life and Vitality in a most indefinte manner, and we look upon all expression or forms of Vital Force as being intensely mysterious and utterly inexplicable ; now while we do not hope to entirely clear up this mystery, we do propose to attempt the removal of some of the obscurity and confusion in which this subject has been so closely enveloped.

To appreciate the full value of this word Vitality, we must analize its applications, and from these analyses, eliminінating all extraneous matter, we can arrive at an approximately accurate idea. By way of illustration, we may say, *first*, grains such as corn, wheat, oats, possess vitality, *second* trees possess vitality, *third*, grass possesses vitality, *fourth*, the herds, which consume the grass, possess vitality ; *fifth*, man, who consumes these herds, possesses vitality. If we proceed to a critical examination of the above statements, we will find that this element which is common to both the animal and vegatable kingdom, can be expressed very readily when the idea of *force* is adopted ; we will call this, "*Vital Force*" for the present.

This force has three forms or modes of manifestation, *first*, *assimilative; second*, *reproductive; third*, *developmental ;* it is very evident that upon these three points plants and animals are equal ; a plant can assimilate food (carbonic acid, ammonia, water, saline matter) can vitalize and incorporate this material, so as to make it a part of itself, and thus it can grow, develope and reproduce its kind ; an insect, an animal, a bird for example, possesses the same forces, and may give us the three expressions of vital force.

Vitality, therefore, as shown by its manifestations of force, has its expression by forms the same in the vegetable as in animal kingdoms, and hence these have received the names of, the "*Vegatative Forces*," the "*Nutritive Forces*," the "*Forces of Organic Life.*"

You will at once appreciate the fact, that as they are active in the vegetable world and as vegetative life is essentially a constructive process, these *forces* must be essentially *constructive.* Indeed their very essence is this construtive action, and we shall find that this nature, so clearly demonstrated in the lower world is found equally as potent in the higher, and the histogenetic forces of the plant are the histogenetic forces of the animal, while the laws and conditions of their exhibition, are at least analogous and parallel if not identical.

If therefore the physical forces, light and heat, acting upon a vegetable germ, will cause the evolution of the vegetative forces, and thus give an exhibition of vitality, will not the same results ensue from these physical forces acting upon the animal germ ?

These forces called vegetative are constructive, and one of the first steps must be the construction of the systems, or machinery of the animal, for they must be constructed before they can be worked ; the organs, tissues &c., must be built or produced before the phenomena of their action can be observed ; we should therefore infer that physical forces would develope in the animal germ exactly the same exhibitions of force that are developed in the vegetable.

To better illustrate this truth, and at the same time give you a practical idea of the power of the physical forces, we will take "Heat" as their representative and pass it first through a vegetable germ and then through an animal germ. The form of force which we call Heat, is the one specially *adapted* to this experiment and its action is interesting in the highest degree.

A grain of wheat, which has lain 3000 years, wrapped in the cerements of an Egyptian mummy, will when supplied with heat and moisture, germinate ; by germination we mean simply to state in ordinary language, that this wheat is still capable of exhibiting the three powers, or three manifestations of vegetable life—it possesses : 1st the Assimilative, (2) Developmental, (3) Reproductive. The Heat acting as a "Dynamical Stimulus" seems to change its form, and reappears as a *Vegetative Force;* it is apparently *lost* as *heat*, but *regained* as *vitality*.

Now in the animal world, we will take as the most appropriate example, "an egg."

The egg is perfectly familiar to you, and yet it will give you a most happy means of illustrating the power of heat. The egg consists of two very different substances: (1) A germ, (2) food for this germ; the whole egg is not a germ—but only a very, very small portion—a mere speck is the true germ; now if we apply a certain amount of heat and apply it continuously for a given period, this force which we call heat, acts upon the *germ* and determines changes in it—and this germ at once exhibits the vegetative forces; it first assimilates a portion of the food or yolk, and thus grows; then it begins to develop, and if the heat be continuously applied, the whole supply of food (i e yolk and white) is assimilated, the developmental force is exhibited, and a living animal is produced.

On the other hand, if the heat be too long withdrawn, or entirely withdrawn, there is a sudden arrest of all vegetative changes—vitality ceases and chemical decompositions ensue; the egg in a short time becomes putrescent. You will please notice, that "Heat" inaugerated the vegetative changes, and that vitality continued as long as this "physical force" was supplied, but ceased upon its withdrawal. The changes were those of nutritive life—organic life—vegetative life; they were begun by heat, continued by heat and ceased when heat was withdrawn; now this looks as if *Heat* passed through an organic gerum may become lost as heat and yet reappear as vegetative force. In other words, a given physical force, *Heat*, may be correlated through an organized structure into vegetative force and reappear as that mysterious thing, *Vitality*.

Vitality then is not to be considered as an entity, as a thing distinct in itself, but as a manifestation of force through organized structure; vitality is inseparable from organization; apart from organization we know nothing of vitality; and

I may as well add, that even with organization we have no knowledge of vitality, save when an organized structure, or organic germ is acted upon by the physical forces; these are the *dynamical conditions* of life.

It has been said that there is in the "grain of wheat," and also in the 'egg,' a *Dormant Vitality*; that may be true, but to us, it is of no practical value, since without the addition of the physical *force heat*, we would have remained ignorant of its dormant presence. It is the physical force, which entering the organism, loses its form of expression as a physical power, and reappears correlated—correlated into vegetative force—correlated into *assimilative, reproductive and developemental force.*

I do not wish to be misunderstood upon this point, so I therefore assert, that I do not say heat and vitality are identical, but I do most positively affirm that so far as this egg is concerned, our knowledge of its vitality depends directly and absolutely upon the fact that *heat* will cause *it* to exhibit the vegetative forces; and just here, I would remind you that the organic germ in the egg, supplied with food available and stimulated by heat, consumes the food, develops a complex organization; constructs systems, organs, tissues; constructs a nervous, a mascular; a circulatory, a respiratory; a digestive system; and brings forth the organic germ, as a complex machine, an animal prepared with all of its systems of animal life in full running order; but notice, it thus developes only when supplied with Heat.

But is this correlation between heat and vitality confined to the embryonic stage of existence? By no means; and here I will give you only two instances, but in a future lecture I will discuss more fully this question of heat and adult life; "if, (says Carpenter,) the Triton (common water newt,) be kept at a temperature between 58° and 75°, it has the power of *reproducing a limb* which has been cut off, but if the temperature be lowered, it loses this reproductive power. And in like manner a snail can regenerate *its head*, if it be kept in a warm atmosphere, but not at a low temperature." In fact so very necessary is heat in adult life, that animals are provided with a special function, the *Calorifacient* or *Heat producing*, by which they preserve an almost uniform temperature; and should this temperature be lowered to any great extent serious results ensue. Therefore, not only is heat necessary to the growth and development of the organic germ, but also to the maintenance of its integrity; and from the vegetable and animal world we gather the evidence of its potent influence, especially in the constructive or histogenetic processes; and as we carefully analize the phenomena of its action and the conditions of vitality, we become more impressed with the truth of the statement that "*Heat by an organic germ is correlated into vitality.*" Now, therefore if heat when passed into an organic germ, reappears as "vegetative force," and if this force continues active for any length of time, and if as stated this force is eminently constructive, the ultimate result of its action must be to *store up force.* When a given amount of *Heat* and its correlative *Light*, have been consumed, and correlated into this constructive force, and when this force ceases to act, have we lost this heat and light? Can we by any known process, recover and make perceptible this stored up force?

The answer comes to us to-day in a very practical form; what is that black substance within that stove? You will answer correctly, "it is coal." But physiologically what is coal? A vegetative production—a result of vegetation; a product of vegetative force; possibly thousands of years ago, Heat and Light were consumed in the growth of the magnificent Flora, which in successive centuries accumulating, upon the earth's surface, finally became buried by volcanic or other convulsive changes of that surface and thus have escaped ultimate chemical action, or complete destruction as we say. Now this coal, this black substance, consumed a given amount of Heat and Light in its growth, and we to day have placed it under conditions such as to compel it to return to us, that same amount of Heat and Light. So you perceive that the physical force passed through an organic germ is not lost, but in the vegetable world may actually be regained in its same form and exact equivalent.

As before stated "force" is continuous and indestructible and in the inorganic world we can only change its form, its manifestations ; and this analysis of the phenomena of vegetative life proves the fact that this truth belongs also to the organic world, at least to that lower world which we call the vegetable.

But just here I will make some statements already foreshadowed if not distinctly asserted, viz : that in the two worlds, the vegetative processes are the same, the expressions of vegetative force are the same, the histogenesis if not absolutely identical is but little different; they both have assimilative, reproductive, developmental expressions ot *force*, and so far as mere vitality is concerned no other manifestation. By these forces the *human* machinery is built, the systems of animal life are evolved, and as the human animal is at birth imperfect, and in reality undeveloped, the same forces operating in embryonic life, are continued into infancy and finally to adult life, when all the machinery of animal life is supposed to be at its acme of perfection, and even then they are retained for repair.

From what has been said, you would infer that the laws and conditions of construction in the plant and animal are the same ; this is not absolutely true ; for an animal possesses *vitality* plus *animality* ; and this animality introduces an entirely new element into the equation ; if you will take the simple act of constructive histogenesis, however, the relation is of the closest kind, there being no very essential difference between the germination of a grain of wheat and the histogenetic changes in the incubating egg ; both are essentially constructive, but as soon as the fact of animality presents itself, and any animal power is evoked, the difference becomes marked, and the element of destruction enters.

*Vitality* is *constructive*, *Animality* is *destructive*. No physical machinery runs without waste and wear, no animal machinery runs without waste and wear, and this element of incessant waste and incessant repair incident upon that waste, brings in the elements of difference and wide separation. The plant is simply constuctive,—a simple aggregation of atoms;—no waste, because the whole functional activity of the different parts of the organism tend to increase; the plant is the type of histogenesis. The animal requires the histogenesis. (1) To construct its systems (2) Repair the waste of these systems, the plant simply grows by histogenesis. In other words the "vegetative forces" in the animal world, (1) Constuct—(2) Repair; and therefore animality depends directly upon vitality, but they are by no means identical; the animal forces are (1) muscular, (2) nerve, (3) brain force, and the integrity of these forces depends upon the integrity of those systems, or machinery which we term—muscular, nerve, and ganglia, and cerebral or brain ganglia, but the integrity of these systems is directly as the "vegetative" or "nutritive" forces of these systems, hence directly dependent upon the constructive vegetative force; for by this force they are *primarilg developed*, and by this their *anatomical exactness* is *preserved*; by this force the wear and waste is repaired, and the *systems are kept in running order.*

That you may the better understand this I will repeat; "*Animality*, as we shall study it in the human being, or even in warm blooded animals where the processes are active, is the direct result of certain systems; the product of the working of the systems; the tissues, organs and systems which give *animality*, constitute a complicated machinery, but the forces by which this machiney is first constructed and afterwards kept in running order, are the vegetative, and thus *animality* while entirely distinct from *vitality* is directly dependant upon it as is stated above." Vitality has reference therefore to histogenesis, animality has reference to the phenomena of *system working*; the tissues &c., which exhibit *animality* have no claim or right to *vitality*, and we shall find in our studies, that the anatomical elements are distinct. That element, anatomically speaking, which constructs the machinery never works as part of this machinery; in fact it is entirely different, from the working element, and is called by several names; Beale calls it *germinal matter*"—Chambers calls it "*nuclear matter*, "Huxley calls it *protoplasm.*

This element is found in all tissues, and necessarily so, as it is the constructive element of these tissues; it is found in muscle, but it is a very different thing from muscular fiber; the fiber is the *animal working element* of the muscle; the fibre under proper conditions will give you muscular force, but it cannot repair itself, whereas the germinal matter cannot give the faintest evidence of muscular force, but it can and does repair aud replace the waste which the fiber has sustained in acting.

You will recall the fact, that vitality is the common heritage of both animal and vegetable kingdoms, and its expression in each is by the three vegetative forces; remember the additional fact, that it is the *germinal matter* which exhibits these powers and that therefore *germinal matter* is the great constructive basis element of organic life. In our next lecture we will discuss Germinal Matter.

